# One-Pot Reducing Agent-Free Synthesis of Silver Nanoparticles/Nitrocellulose Composite Surface Coating with Antimicrobial and Antibiofilm Activities

**DOI:** 10.1155/2021/6666642

**Published:** 2021-03-27

**Authors:** K. G. U. R. Kumarasinghe, W. C. H. Silva, M. D. A. Fernando, L. Palliyaguru, P. S. Jayawardena, M. Shimomura, S. S. N. Fernando, T. D. C. P. Gunasekara, P. M. Jayaweera

**Affiliations:** ^1^Dept. of Chemistry, University of Sri Jayewardenepura, Nugegoda, Sri Lanka; ^2^Research Institute of Electronics, Shizuoka University, 3-5-1 Johoku, Naka-ku, Hamamatsu, Japan; ^3^Dept. of Microbiology, University of Sri Jayewardenepura, Nugegoda, Sri Lanka

## Abstract

Nitrocellulose with silver nanoparticle (AgNP/NC) composite was prepared *in situ* using Ag(CH_3_CO_2_) and nitrocellulose without any reducing agent. The composite materials synthesized were spray coated onto glass substrates to obtain thin films. The AgNPs/NC composites were characterized by ultraviolet-visible, Fourier transform infrared, X-ray photoelectron spectroscopy, scanning electron microscopy, and transmission electron microscopy. The antimicrobial activity of AgNPs/NC composite was investigated by tube method and time-kill kinetic studies against three microbial species, including *Pseudomonas aeruginosa* (ATCC 27853), *Staphylococcus aureus* (ATCC 25923), and *Candida albicans* (ATCC 10231). The antibiofilm activities were qualitatively determined against all three organisms. Prepared AgNPs/NC films exhibited good antimicrobial activity and significant inhibition of biofilm development against all three microbial species. The effective dispersion of AgNPs/NC in biofilm was responsible for the significant antibiofilm activity of the prepared material. The reported AgNPs/NC composite can be used as coating additive in bacteriocidal paint which can be applied onto surfaces such as in healthcare environments.

## 1. Introduction

There are increasing reports of patients with hospital acquired infections and emergence of multidrug-resistant microbial pathogens leading to a higher mortality, morbidity, and increased length of hospital stay [1–3]. Microorganisms that attach to the surfaces in the healthcare environment, such as hospital beds and walls, can cause acute and chronic infections [4, 5]. These bacteria or yeast form multicellular biofilms in which the microorganisms are protected from killing by host defenses and antibiotics [6]. Contamination of surfaces such as walls and beds especially in the hospital setting can transmit infection via hands. For example, in operation theatres, this poses a high risk of surgical site infections, which can be transmitted to the patient via contaminated surfaces [7, 8]. Thus, the surface contamination has been shown to be directly proportional to patient to patient transmission of nosocomial infections [9]. Centers for Disease Control and Prevention guidelines recommend use of several chemical disinfectants to achieve low-level and high-level disinfection. These include glutaraldehyde, ethyl or isopropyl alcohol, sodium hypochlorite, hydrogen peroxide, and phenolic detergents. Further, the establishment of biofilms on environmental and biological surfaces results in persistent foci of infection. Therefore, treatment of susceptible inanimate surfaces with an antimicrobial coating could be useful in the prevention of biofilm formation [6, 10]. The development of healthcare-related products, such as textiles, medical devices, and surface coatings, with antimicrobial and antibiofilm properties can significantly reduce transmission of microbial infections [11–14].

Development of surface coatings with antibacterial properties is paramount in healthcare environment. Investigations to develop low cost, simple, and novel surface coatings with antimicrobial properties are an urgent need. Here, we describe a low cost, one-pot method to synthesize silver nanoparticle nitrocellulose (AgNP/NC) composite as a potential antimicrobial surface coating, which can be applied to public and healthcare environments.

Nanomaterials have attracted significant attention as antibacterial agents, especially in the development of medical applications [15]. For instance, many nanoparticles, such as metals (Ag and Au) and metal oxides (ZnO, CuO, TiO_2_, and MgO), have provided numerous applications in the biomedical field [16–18]. The applications of nanomaterials with antibacterial properties are not limited to therapeutic applications, but also useful for the development of antibacterial products such as coatings and water filters [14, 19]. Nanoparticles can be effectively functionalized or impregnated with suitable matrix, allowing them to offer a class of advanced materials for various commercial products for the prevention of bacterial infections [20]. Among them, AgNPs possess exceptional antibacterial activities [21]. Silver-based compounds including AgNPs are known to be biocidal for a vast range of bacteria, which includes *E. coli* [22]. A wide variety of methods have been developed for the synthesis of AgNPs. Among those, the green synthesis of AgNPs is gaining more attention among researchers in recent years because of its advantages, such as ecofriendly, simple, nontoxic, and economical over traditional chemical and physical methods [23]. In the green synthesis, reducing agents such as various plant extracts, fungi, and bacteria are used to reduction of Ag^+^ ions to Ag in aqueous and nonaqueous solution [24, 25]. Use of silver nanoparticles enhances the contact between microorganisms and the metal nanoparticles due to the high surface to volume ratio [26]. Kim and coworkers have shown that AgNPs show similar inhibitory properties against *E.coli* and yeast while the inhibition is comparatively lesser against *S. aureus* [22]. AgNP-based biomaterials, such as ointments, catheters, and antibacterial textile fabrics, are commercially available and actively utilized in healthcare systems worldwide [27, 28]. Nano-enhanced paints and coatings were developed by various researches to enhance or fabricate the functionalities of paint varieties and/or coatings such as decorative appearance, protection against radiation, humidity, microorganisms, fire protection and thermal insulation, scratch resistance, and UV-protection [29, 30].

NC is one of the most commonly used compounds and most important cellulose derivative in paint industries [31]. It is basically used as binder in paint formulations. Moreover, many materials commonly used in daily life, such as pharmaceuticals, printing inks, and decorative films contain NC [32]. There are various methods developed by researchers for the incorporation of metals to polymeric matrices such as chemical reduction reactions, mixing preformed metal nanoparticles with polymers, and complicated physical techniques, such as sputtering, plasma deposition, and layer-by-layer deposition [14, 33–36]. These methods contain many disadvantages such as time consumption and cost.

To the best of our knowledge, no studies have been conducted to investigate the potential of silver nanoparticle NC composite as an antimicrobial surface coating. Nguyen and coworkers reported NC-stabilized silver nanoparticles can be used as low conversion temperature precursors useful for inkjet-printed electronics [37]. Another study reported the development of NC membrane filters impregnated with different biosynthesized silver nanoparticles applied to water purification [19]. Development of potential surface coating for effective management of hospital-acquired infection, with a low cost and ecofriendly method using NC and Ag nanoparticles, is described in this paper.

## 2. Materials and Methods

### 2.1. Preparation of AgNPs/NC Composite

Samples of commercial grade ½ sec RS nitrocellulose (molecular weight 999.4 g mol^−1^ and nitrogen content ~11 wt%) were gifted from Nippon Paints (Pvt) Ltd, Sri Lanka, and used as received. In a typical experiment, 10 mL from saturated solution of Ag(CH_3_CO_2_), (Silver Acetate Extra Pure, molecular weight 166.9 g mol^−1^, Techno Pharmchem) in methanol was added dropwise to solution of NC (2.5 g) dissolved in 10 mL ethyl acetate AR (molecular weight 88.1 g mol^−1^, Techno Pharmchem). The mixture was continuously stirred at 60°C (Velp Scientifica AREC heating magnetic stirrer, ~300 rpm) and turned into dark orange color which takes about two hours. For the preparation of film, AgNPs/NC solution was spray coated (Placehab 0.2 mm Stainless Steel Mini Paint Spray Gun Pen using commercial N_2_ gas, purity ~98%) manually onto glass substrates (60 mm petri dishes, surface is 21.5 cm^2^, Fisher Scientific) and kept at room temperature for overnight.

### 2.2. Characterization Methods

Optical properties of AgNPs/NC were characterized by a UV-Vis spectrophotometer (PerkinElmer Lambda 35) in the range of 350–700 nm. ATR-FTIR analysis (Nicolet iS10 FT-IR spectrometer) was carried out in a spectral range of 400–4000 cm^−1^, resolution of 4 cm^−1^, and using the OMNIC spectra™ data processing software. The morphology and size distribution of the AgNPs/NC were analyzed using scanning electron microscope (SEM, SEM, Zeiss EVO 18) with 3.00 kV accelerating voltage and 8.0 mm working distance and transmission electron microscope (TEM, HR-TEM ZEISS Libra 200 Cs-TEM), respectively. X-ray photoelectron spectroscopy (XPS) analyses were carried out with Axis Ultra DLD spectrometer (Kratos) and monochromatic Al K*α* source. The instrument base pressure was 8 × 10^−10^ Torr. The XPS spectra were collected using an analysis area of ~300 *μ*m × 700 *μ*m. Pass energies of 160 eV and 20 eV were used for wide and narrow spectra, respectively. The charge neutralizer system was used for all analyses. Curve fitting of raw data was performed by using the XPS Peak Fit software Version 4.1 (Morton, 1995–2006) with a 40% Lorentzian/Gaussian ratio and a Shirley background. Thermogravimetric analysis (TGA) and Differential analysis (DTG) were carried out with TA SDT 650 thermogravimetric analyzer under nitrogen atmosphere at a heating rate 10°C min^−1^. Physical parameters important in surface applications were estimated with the support of Nippon Paints (Pvt) Ltd, Sri Lanka. The Krebs Stormer Viscometer (PCE-RVI 6) was used to acquire common paint parameters. The dry to touch time of NC lacquer and NC/AgNPs lacquer was measured using AB3600 TQC Drying time recorder.

### 2.3. Antibacterial and Antibiofilm Activities of AgNPs/NC Film

#### 2.3.1. Tube Method

The antimicrobial activity of NC and AgNPs/NC strips against *Pseudomonas aeruginosa* (ATCC 27853), *Staphylococcus aureus* (ATCC 25923), and *Candida albicans* (ATCC 10231) was determined using the tube method. Each organism was cultured overnight at 37°C in nutrient agar. Two loops full of each pure culture were used to inoculate 30 mL sterile nutrient broth and incubated at 37°C overnight. The suspension was then diluted 1 : 100 into fresh nutrient broth medium containing 1 wt% dextrose and adjusted to 0.5 McFarland turbidity standard. Five hundred microliters (500 *μ*L) of suspension was added to each tube.

Sterile NC strips (negative control) and AgNPs/NC strip (test) having a dimension of 0.5 cm × 1.5 cm were prepared by cutting with a sterile scissor. All the strips were immersed in 70% (v/v) ethanol for 30 seconds and allowed to dry in the biological safety cabinet. The negative control tube was prepared by inserting a NC strip to uninoculated nutrient broth, and the positive control tube was prepared by inserting a NC strip to the inoculated nutrient broth, respectively. AgNPs/NC strip inserted to an inoculated nutrient broth was used as the test sample. All tubes were incubated overnight at 37°C. To determine the antimicrobial activity, a viable cell count by spread plate method and microbial adhesion to the strip was evaluated. In brief, to obtain the viable cell counts, aliquots of the broth were used to prepare tenfold serial dilutions and 100 *μ*L was spread on sterile nutrient agar plates. These plates were incubated at 37°C for 24 h for the bacterial species and 48 h for *C*. *albicans* before taking a viable cell count. The average reduction percentages of colony-forming units (CFUs) were calculated using the following equation [38]:

(1) $$\begin{array}{c} \text{Average reduction }\left(\%\right)=\frac{\text{CFU in ml of control}–\text{CFU in ml of test sample }}{\text{CFU in ml of control }}\times 100. \end{array}$$

To determine the microbial adhesion on strips treated with AgNPs, the strips were gently washed thrice with sterile phosphate buffered saline to remove any nonadherent cells. Excess solution was removed by touching the edge of a strip to a sterile filter paper to drain excess moisture. The strip was then transferred to the surface of a sterile nutrient agar plate and incubated at 37°C overnight. The plates were observed after 24 h for bacteria and 48 h for the *C. albicans*, and any growth around the strips were recorded. All experiments were carried out in triplicate.

#### 2.3.2. Time-Kill Assay by Plate-Coating Method

The plate-coating method was conducted using three microbial strains: *Pseudomonas aeruginosa* (ATCC 27853), *Staphylococcus aureus* (ATCC 25923), and *Candida albicans* (ATCC 10231). Sterile petri dishes with a diameter of 6 cm were spray coated with 2 mL NC alone or AgNPs/NC solution. A negative control plate was prepared by adding 2 mL sterile distilled water. The plates were allowed to dry completely for 24 h inside a class II biological safety cabinet. Afterward, 2 mL of standard bacterial suspension (0.5 McFarland standard) prepared in sterile nutrient broth was added to each petri dish and incubated at room temperature for different time intervals. Aliquots of 100 *μ*L of the bacterial suspension were collected at different time intervals and spread on sterile nutrient agar plates. These plates were incubated at 37°C for 24 h for culture of bacterial species and 48 h for the culture of *C*. *albicans* before taking a viable cell count. Experiments were triplicated for each microbial species [38]. The average percentage reduction of colony-forming units (CFUs)/mL obtained at different time intervals were calculated using the above equation.

## 3. Results and Discussion

### 3.1. Optimization of AgNPs/NC Synthesis

**Figure 1:**
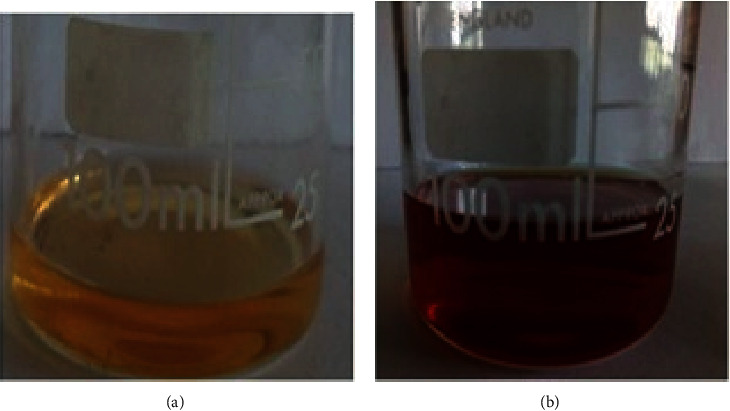
NC ethyl acetate mixture heated at 60°C for 1 h in the (a) absence of and (b) presence of Ag ions.

In the preparation of AgNPs/NC composite, a pale red coloration characteristic (Figure 1) to AgNP formation was observed within ~15 min after the addition of Ag(CH_3_CO_2_)/methanol mixture to a NC dissolved in ethyl acetate at elevated temperatures (40°C-60°C) with stirring at ~300 rpm. Upon heating at 40°C as well as at 60°C, the pale red color was further enhanced and reached its maximum optical density approx. 180 minutes.

**Figure 2:**
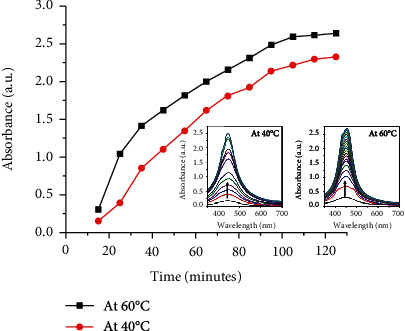
SPR peak intensity variation with reaction time at 40°C and 60°C; UV-Vis absorption spectra. NC to Ag ratio 1.0 : 0.05 (wt%).

UV-Vis absorption spectra recorded with time for two different temperatures, 40°C and 60°C having a NC to Ag ratio (wt%) at 1.0 : 0.05, are shown in Figure 2. The characteristic surface plasmon resonance (SPR) peaks appear at 440 nm and 450 nm for AgNPs/NC solutions prepared at 40°C and 60°C, respectively. Further, Figure 2 shows that absorption maxima reach the saturation in ~120 min of reaction time, indicating the completion of NP formation.

**Figure 3:**
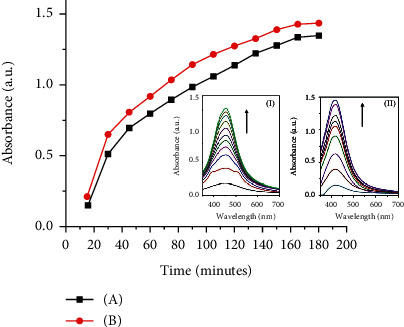
SPR peak intensity variation with reaction time at (a) 40°C and (b) 60°C; UV-Vis absorption spectra (I) 40°C and (II) 60°C; NC to Ag ratio 1.0 : 0.05 (wt%).

Higher reaction temperatures, i.e., >60°C, were not attempted due to high evaporation losses of ethyl acetate/methanol solvent mixture. The mixture was allowed to cool to room temperature with vigorous stirring to get homogeneous syrupy liquid of AgNPs/NC. Two silver ion concentrations were tested with NC to Ag mass ratios (w/w) 1.0 : 0.05 and 1.0 : 0.10 in this work. Higher concentrations of Ag^+^ in the reaction medium enhance the rates of formation AgNPs/NC composite (Figure 3). However, addition of excess amounts of Ag^+^ causes to AgNPs/NC solution to become turbid and films to be nontransparent and brittle. Hence, throughout this work 1.0 : 0.10 mass ratio was selected as the optimum Ag concentration (1.24 × 10^4^ nM). Thin films of AgNPs/NC composite were prepared by spray coating the syrupy liquid onto glass substrates using a solvent mixture ethyl acetate/methanol/butylglycol (7 : 7 : 1 volume ratio) as the diluting agent.

**Figure 4:**
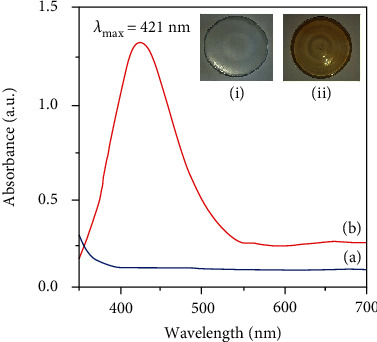
UV-Vis absorption spectra (a) NC film and (b) AgNPs/NC film. Images of (i) NC and (ii) AgNPs/NC films coated onto petri dishes.

The UV-Vis absorption spectrum of the film of AgNPs/NC (thickness ~200 *μ*m) composite shows the absorption band centered at 412 nm (Figure 4(b)), which is characteristic to the SPR of AgNPs. Presence of AgNPs in the NC film imparts an off-yellowish color to the film (Figure 4(ii)). The absence of Ag in the control experiment resulted in a clear transparent NC film (Figure 4(i)) and showed no absorption bands in the region of 300-700 nm (Figure 4(a)). Appearance of SPR peak for the AgNPs/NC film is a strong indication for the presence of AgNPs in the NC matrix. Further, a narrow SPR peak with a FWHM (full width at half maximum) of 105 nm indicates fairly a narrow particle size distribution.

**Figure 5:**
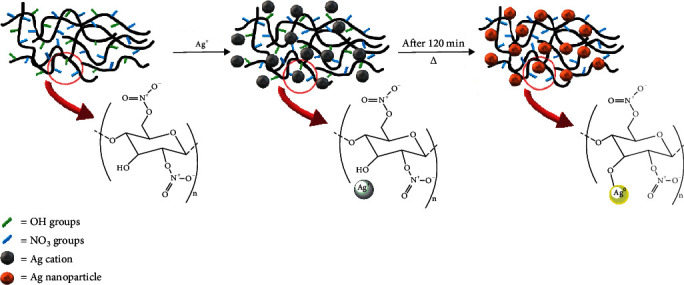
Representation of the *in situ* synthesis of AgNPs on NC polymer matrix.

Surface stabilization agents prevent nanoparticle aggregation and organic polymers have attracted much attention due to their high functionality along the backbone, with side groups attached to the backbone or with pendant side chains [39]. Polymers containing thiol, amine, hydroxyl, carboxylic acid, and ester groups have demonstrated the ability to stabilize Ag, Au, and Pt nanoparticles in organic and aqueous media [40–43]. Cellulose and its derivatives have been widely employed in the synthesis of AgNPs for various applications [44, 45]. The reduction of Ag ions followed by stabilization of Ag^0^ clusters against agglomeration is essential in the AgNP preparation process. In the *in situ* preparation of AgNPs using NC as the matrix, silver ions (Ag^+^) are distributed in the NC fiber matrix by forming a complex between Ag^+^ and hydroxyl groups present in the commercial grade NC (Figure 5) [19, 46]. This is possible as nitrogen content is 10-11 wt% in the commercial grade NC. The degree of NO_2_ substitution to the cellulose moiety is mostly as di- and mononitrates with one or two OH groups in the cellulose unit allowing to form AgNPs [47, 48].

### 3.2. Kinetics of AgNPs/NC Composite Formation

A higher rate for the formation of AgNPs was observed at an elevated temperature of 60°C, which is in agreement with the increased reducibility of hydroxyl groups in cellulosic materials reported in previous studies [49]. Silver nanoclusters formed within the matrix are confined to the sites, where hydroxyl groups are located within the NC matrix and therefore stable against the agglomeration.

The SPR peak intensity of the reaction mixture was employed to investigate the kinetic parameters of NP formation at two different temperatures and NC : Ag mass ratios. AgNPs/NC composite formation was studied under excess molar amount of NC with respect to Ag. Therefore, one can assume that NP formation proceeds under pseudo-first order reaction kinetics. The concentration of Ag ions at a given time is inversely proportional to the extent of AgNPs in the solution, assuming at all Ag ions are used for the formation of AgNPs. The extent of AgNPs in the solution is directly proportional to the SPR peak intensity. Hence, the extent of AgNPs at a given time was calculated by [50].

(2) $$\begin{array}{c} \left[\text{AgNP}\right]\%=\frac{S\text{PR peak intensity at tim}{\text{e}}^{"}t^{"}}{\text{maximum SPR peak intensity}}\times 100 \end{array}$$

where *t* is elapsed time. Therefore, Ag^+^ concentration was determined by

(3) $$\begin{array}{c} \left[\text{A}{\text{g}}^{+}\right]\%=100-\left[\text{AgNP}\right]\% \end{array}$$

**Figure 6:**
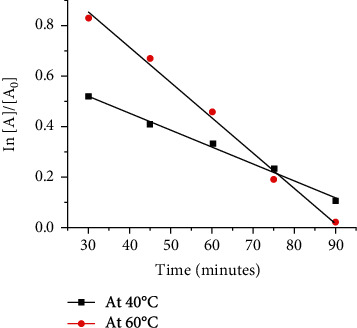
Pseudo-first order reaction kinetic plots for the reduction of Ag ions at 40°C and 60°C.

The natural logarithm of the Ag^+^% decreases linearly as a function of time (Figure 6) confirming the pseudo-first order reaction kinetics for the reduction of Ag^+^ ions in the NC matrix.

The rate constants for the reduction of Ag^+^ for the mass ratio 1.0 : 0.10 (NC : Ag) at 40°C and 60°C are reported in Table 1. Values were calculated from the slope of the linear fit. The reduction rate constant increases from 6.0 × 10^−3^ min^−1^ to 14.0 × 10^−3^ min^−1^ with increasing temperature from 40°C to 60°C. The kinetic data under varied temperature conditions shows the input of additional energy has increased the reducibility of Ag^+^ by NC. This finding is also in agreement with previous attempts on *in situ* formation of AgNPs in cellulosic materials [51].

### 3.3. Surface Characterization

**Figure 7:**
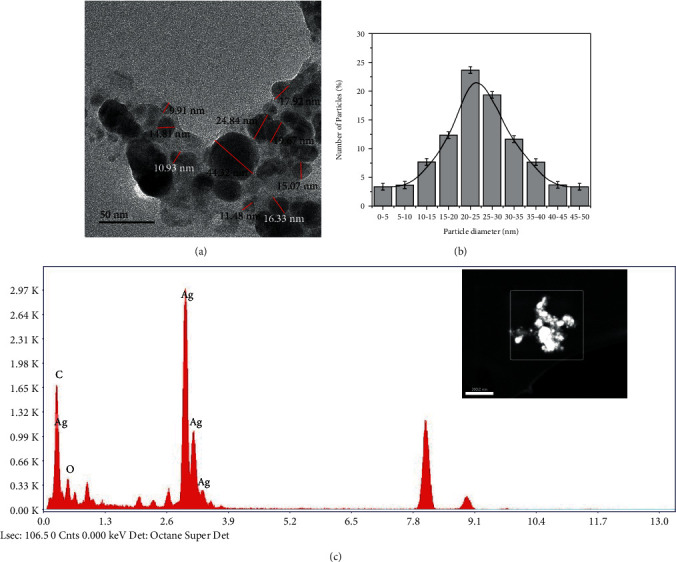
TEM image of (a) AgNPs/NC, (b) histogram of particle size distribution, and (c) EDX analysis.

**Figure 8:**
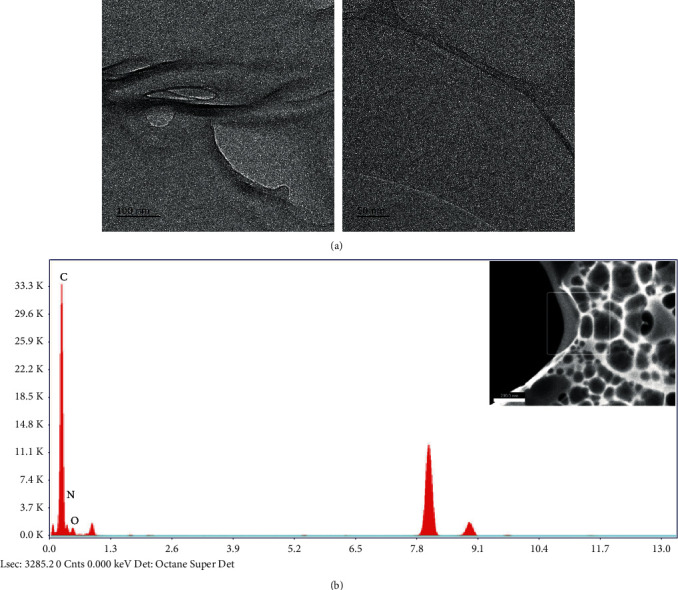
TEM image of (a) bare NC sample and (b) EDX analysis.

Surface morphology of AgNPs/NC composite and NC were investigated with SEM and TEM images.  Figure 7(a) shows a TEM image of AgNPs/NC composite prepared at 40°C with 1.0 : 0.10 NC to Ag mass ratio. Image clearly indicates the presence of spherical nanoparticles that are fairly well segregated by NC matrix. The histogram of size distribution created by image analysis (Figure 7(b)) shows 90% of the nanoparticles are in the range of 5-50 nm with an average particle size of 25 ± 5 nm. The TEM image of a bare NC sample is used as the control (Figure 8).

**Figure 9:**
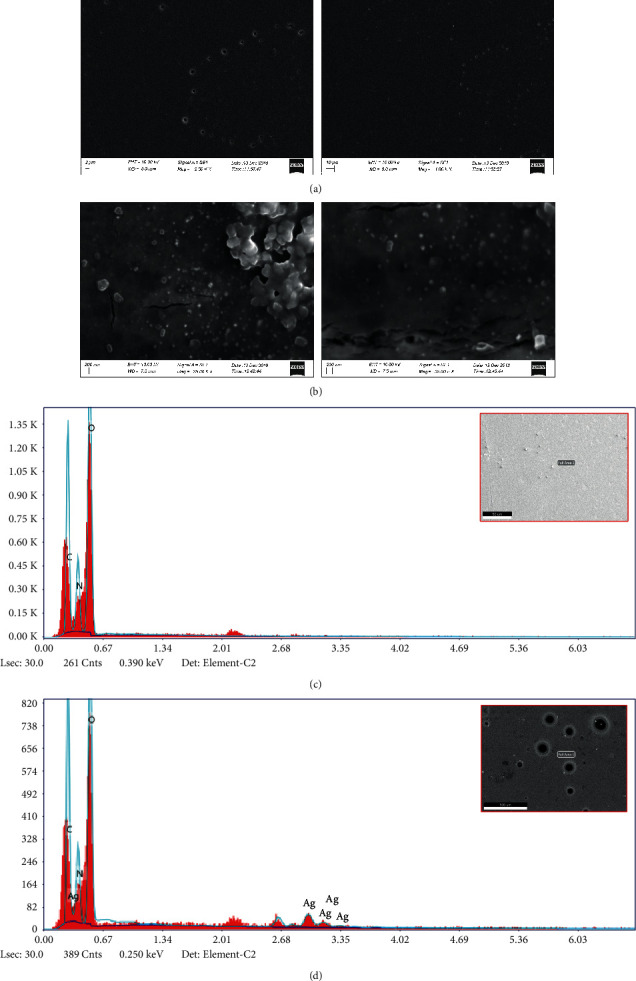
SEM images of film of (a) bare NC and (b) AgNPs/NC sample; EDX analysis (c) bare NC and (d) AgNPs/NC sample.

The EDX analysis data of AgNPs/NC are shown in Figure 9(c). The presence of silver in the composite is evident by the strong peak appearing at 3 keV region in the spectrum [19]. The EDX determined the Ag atom percentage was 0.15%. The absence of such intense peak is evident for the bare NC (Figure 8). The SEM images obtained for NC and AgNPs/NC thin films also indicate smooth, spherical AgNPs with size in the range of 20 nm to 80 nm. The nanoparticles are fairly monodispersed in the NC matrix. Even in areas where aggregates are visible, nanoparticles do not show direct contact with these aggregates. These facts confirm the ability of NC to act as a potential stabilizer. EDX analysis further confirms the presence of Ag in the NC matrix (Figure 9). The cracks observed in the AgNPs/NC (Figure 9(b)) may be due to the thermal damage induced by the high-impact electron beam during SEM analysis.

**Figure 10:**
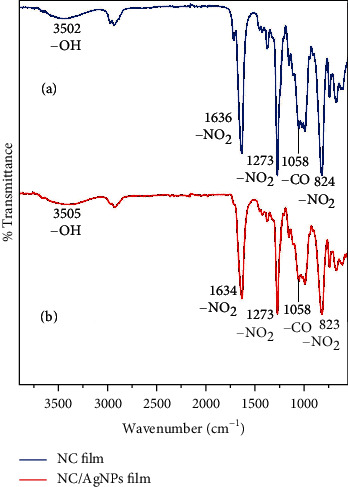
ATR-FTIR spectra of the prepared films (a) NC and (b) AgNPs/NC.

FTIR spectroscopic analysis was carried out to investigate the functional groups involved in the reduction and stabilization of AgNPs (Figure 10). The FTIR spectra of the prepared films, i.e., NC and AgNPs/NC, show three intense peaks at around 1634 cm^−1^, 1273 cm^−1^, and 823 cm^−1^, indicating the presence of nitrate groups. At around 3503 cm^−1^, a broad peak appears for both films indicating the presence of OH groups. When compared, it is clearly visible that the FTIR spectra of NC and AgNPs/NC composite films do not show significant differences, indicating NO_2_ groups are not playing a major role in the formation of NPs. Furthermore, solid matrix may have caused steric impediments thus making it difficult to clearly observe the changes in the FTIR spectra which would have arisen due to the chemical interaction between NC and silver nanoparticles [52].

**Figure 11:**
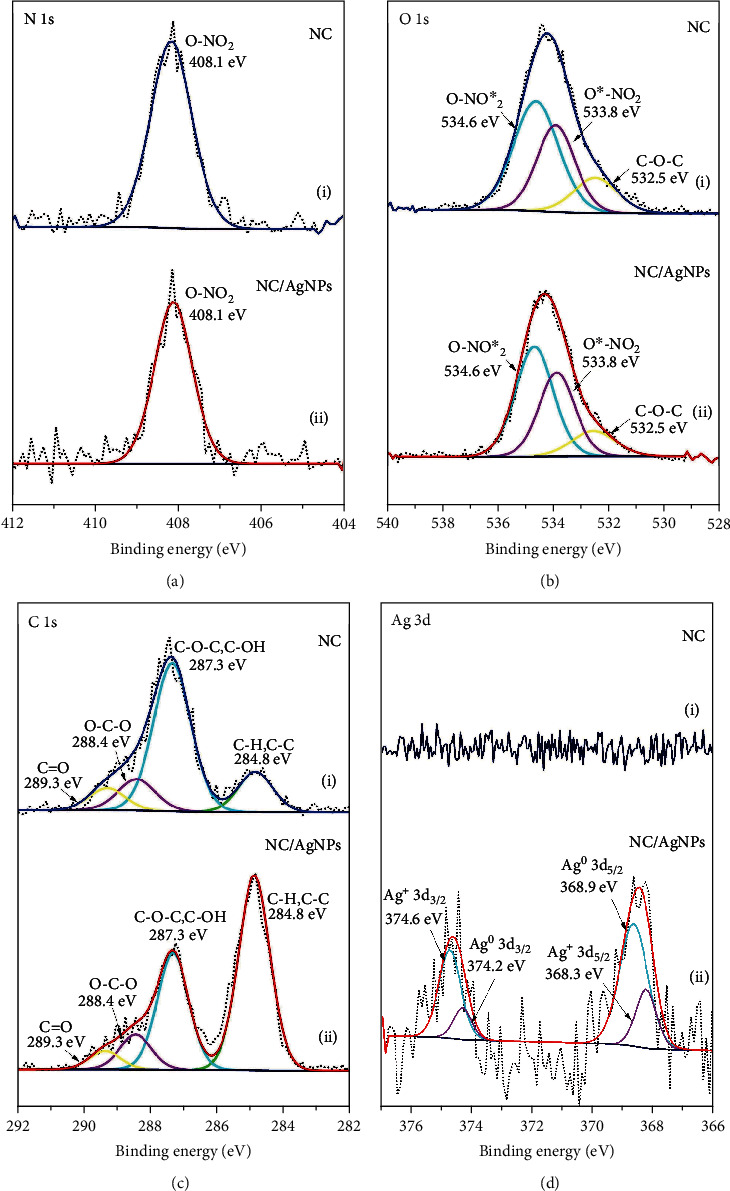
High-resolution XPS scans of NC and AgNPs/NC: (a) N 1 s, (b) O 1 s, (c) C 1 s, and (d) Ag 3d regions.

High-resolution XPS scans recorded for C 1 s, O 1 s, N 1 s, and Ag 3d regions of NC and AgNPs/NC are shown in Figure 11. Based on previous work [53, 54] C 1 s peaks located at 284.8 eV, 287.3 eV, 288.4 eV, and 289.3 eV can be assigned to -C-C/-C-H, -C-O-C-/-C-OH, O-C-O, and –C=O, respectively. The observed relative intensity variation of peaks at 284.8 eV and 287.3 eV may result upon AgNP formation through the –C-OH where Ag nanoclusters are stabilized via NC hydroxyl groups. O 1 s scans were curve fitted for three features with binding energies of 532.5 eV, 533.8, and 534.6 eV. Peaks were ascribed to –C-O-C, -O^∗^-NO_2_, and -O-NO_2_^∗^ in the NC. The XPS spectrum of N 1 s region for both AgNPs/NC and NC shows symmetric peak at 408.1 eV which can be assigned to the nitrogen in –O-NO_2_. The peaks appearing in the binding energy range of 357-362 eV in the XPS spectrum for AgNPs/NC are due to Ag° 3d_3/2_ and Ag° 3d_5/2_ at 374.6 eV and 368.9 eV, respectively, with a spin-orbit separation of about 6.0 eV for Ag 3d [55]. The peak fitting confirms the presence peaks corresponding to Ag^0^ and in addition to peaks at 374.2 eV and 368.3 eV corresponding to Ag ion. A fraction of Ag ions could be present in the form of Ag_2_O in the NC matrix as it has been reported in similar studies. The formation of metallic Ag and Ag_2_O are possible when reactions are performing in ambient conditions [55]. The Ag atom percentage resulted from XPS was 0.27%.

### 3.4. Stability and Properties of AgNPs/NC Films

**Figure 12:**
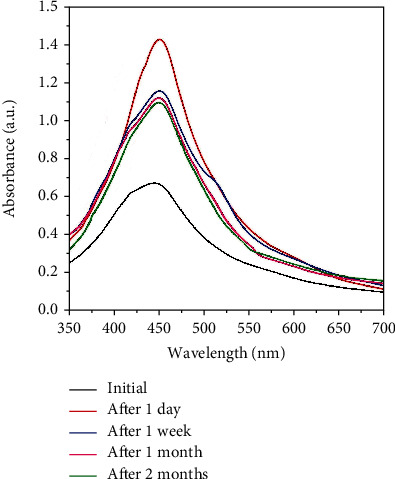
UV-Vis absorption spectra of AgNPs/NC film with time under ambient conditions.

The stability of AgNPs/NC film is an important criterion for any practical application. Therefore, the stability of AgNPs/NC film under ambient conditions was tested by studying the SPR peak pattern with time (Figure 12). The SPR peak does not show a significant peak shift despite that its intensity varies from spectral scans. The variation of SPR intensity from one scan to another is due the thinness variations of the AgNPs/NC film [56–58]. The absence of a shift suggests that AgNPs formed within the NC matrix poses the stability against the agglomeration. A visual inspection of the AgNPs/NC film carried out over the same period did not show any noticeable change in appearance.

**Figure 13:**
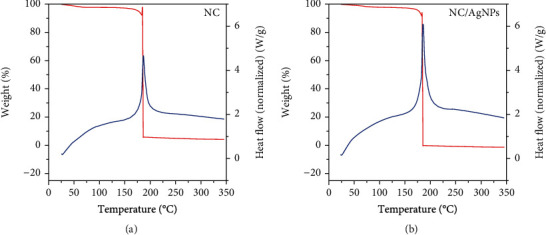
TG and DTG curves of (a) NC and (b) AgNPs/NC film.

Thermal decomposition behaviors of the AgNPs/NC composite material were also investigated by TG under nitrogen atmosphere. The TG and DTG curves for NC and AgNPs/NC are shown in Figure 13. For all samples, small weight losses of ~5 wt% below 160°C are resulted from the release of bound water from the NC film. A rapid weight loss above the 160°C occurs for both samples due to the initiation of thermal decomposition. Therefore, inclusion of silver bears no adverse effect on the thermal stability of NC film, which is in agreement with the previously published data [47].

Furthermore, surface coating properties of clear commercial (Nippon Paint, Sri Lanka) NC lacquer and the AgNP composite prepared with the same were tested. A 75-micron wire bar applicator with BYK-Gardner drawdown cards was used to acquire common paint parameters. Data presented in Table 2 clearly indicate that there is no major impact on important parameters upon incorporation of Ag, except the color of the AgNPs/NC composite.

### 3.5. Antimicrobial and Antibiofilm Activities of AgNPs/NC

#### 3.5.1. Tube Method

The cell viability assay was used to determine the reduction in colony-forming units/mL in the presence and absence of AgNPs/NC. The average percentage reduction of colony-forming units (CFUs) in the broth with and without AgNPs strip for each species; *P*. *aeruginosa*, *S*. *aureus*, and *C*. *albicans* were determined as 84.3%, 100%, and 92.2%, respectively. These results suggest that in the presence of the AgNPs/NC strip, the bacterial cell viability was clearly decreased.

The mechanism involved in microbial destruction by AgNPs involves contact killing and killing mediated by the action of silver ions diffused out into the environment [59]. AgNPs can attach to the bacterial cell wall and infiltrate the cell reaching the cell membrane. [60] Contact with cell membrane leads to physical disruption of the membrane resulting in cell lysis. On entering the cytoplasm, AgNPs interact with biomolecules and cellular structures resulting in bacterial dysfunction and death [61]. The large surface to volume ratio at the nanoscale enhances its biological activity [61]. Further, AgNPs can generate reactive oxygen species (ROS) which are toxic to bacteria [62].

In this study, AgNPs/NC composite strips, from where Ag^+^ are released into the surrounding environment. Ag^+^ released by AgNPs are also responsible for antimicrobial activity. AgNPs with high surface to volume ratio could release high Ag^+^ concentration and thus have higher antibacterial properties [63]. These ions can interact with sulphydryl groups on proteins and enzymes and result in protein deactivation [63]. Further, Ag^+^ can intercalate between the purine and pyrimidine base pairs preventing cell division [60]. Thus, the high percentage reduction in the test sample which contained AgNP/NC could be due to various mechanisms described above resulting in antimicrobial activity.

#### 3.5.2. Inhibition of Microbial Adhesion and Growth

**Figure 14:**
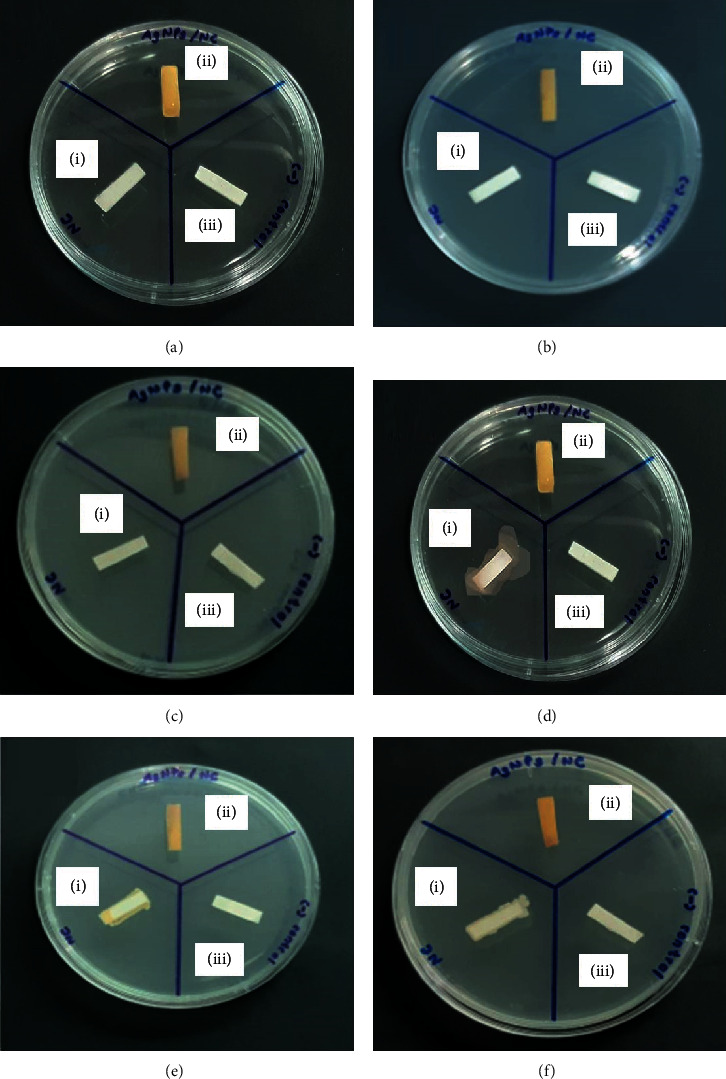
Inhibition of microbial growth on NC strips with and without AgNPs: NC strips labelled as without AgNPs (i), with AgNPs (ii), and in sterile nutrient broth (iii). (a–c) Strips before incubation with test organisms; (d–f) strips after incubation for 24 h with (a, d) *P*. *aeruginosa*, (b, e) *S*. *aureus*, and (c, f) *C*. *albicans* microbial species.

Figures 14(a)–14(c) represent strips before incubation with test organisms and Figures 14(d)–14(f) represent strips after incubation for 24 h. Culture of the AgNPs/NC strips (test sample) resulted in no visible growth around the AgNPs/NC strips as shown in Figure 14 depicted as strips (ii) in all bottom plates. The NC strips without AgNPs depicted as strips (i) had abundant microbial growth around the strip suggesting that the NC substrate promotes adherence of test species to its surface. The negative control strips, which were in sterile nutrient broth (strips (iii)), did not show any growth confirming the sterility of the strips. Thus, the results strongly suggest that incorporation of AgNPs into the NC strip effectively prevents surface adhesion and promotes microbial destruction.

The test species were cultured in medium supplemented with dextrose to promote the growth of bacterial biofilms. The first step in biofilm formation is adhesion. Following attachment, bacteria can multiply and secrete extracellular matrix in which the vegetative cells get embedded [64]. In this study, microbial adhesion was clearly visible in the NC strips without AgNPs, while in the presence of AgNPs, no microbial growth could be detected, which positively suggests that AgNP/NC did not favor formation of biofilms. Thus, it could be useful as a coating to prevent biofilm formation on environmental surfaces. AgNPs have been used as antimicrobial coatings in various medical devices such as catheters, pacemakers, and implants specially due to its ability to prevent biofilm formation [65].

#### 3.5.3. Time-Kill Assay by Plate-Coating Method

**Figure 15:**
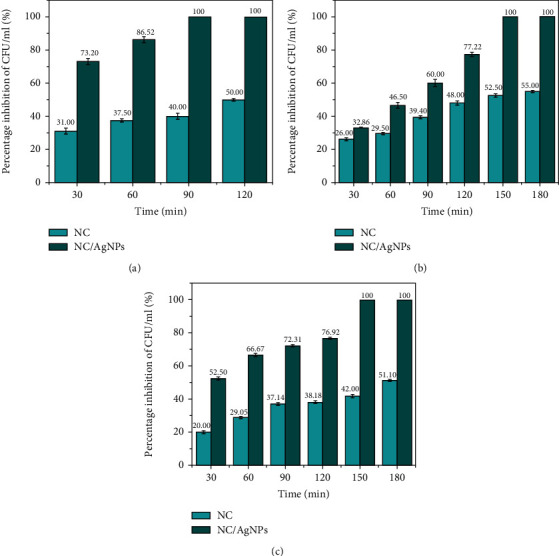
Time-kill assay to determine the contact time required for 100% inhibition of CFU in the test microorganisms. Bar charts represent the percentage inhibition of CFU/ml over contact time of (a) *P*. *aeruginosa*, (b) *S*. *aureus*, and (c) *C*. *albicans* microbial species.

The time-kill kinetic studies were performed to determine the direct contact time that resulted in 100% inhibition of CFUs/mL for each microbial species. The time taken to reach 100% inhibition for test organisms was found to vary (Figure 15). The AgNPs/NC completely inhibited the growth of *P*. *aeruginosa* in 90 min (Figure 15(a)) while the *S*. *aureus* (Figure 15(b)) and *C*. *albicans* (Figure 15(c)) species were inhibited after 150 min of direct contact time. The cell wall and membrane components of Gram-positive bacteria, Gram-negative bacteria, and fungi vary which in turn affects the antimicrobial potential of AgNPs [66].

The cell wall of Gram-negative bacteria is thinner and composed of lipopolysaccharides, lipoproteins, and phospholipids. In contrast, the cell wall of Gram-positive bacteria includes a thick layer of peptidoglycan, teichoic acid which can impede the penetration of the nanoparticles [66]. Further, Gram-positive bacteria have a high negative charge on the cell wall surface, which can attract AgNPs and eventually leads to disruption of cytoplasmic membrane and ultimately cell death [66]. In *C*. *albicans*, the cell wall is typically a layered structure and composed mostly of glucans, chitin, and mannoproteins. Glucans and chitin, the main structural components that supply rigidity to the wall structure, concentrated more in the inner cell wall layer, and the external cell wall layer has consisted abundant mannoproteins which exhibit a fibrillar appearance [67]. A study by Peiris et al. demonstrated that AgNPs had stronger antimicrobial activity against Gram-negative bacteria. In their study, Gram-positive bacteria *S. aureus* required longer contact time (270 minutes) to achieve 100% inhibition of viability compared to *P. aeruginosa* (60 minutes) [68]. The findings of this study are similar as the Gram-negative test strain *P. aeruginosa* demonstrated 100% inhibition at 90 minutes compared to *S. aureus* and *C. albicans* which required 150 minutes of contact for 100% inhibition.

## 4. Conclusions

One-pot reducing agent-free method was developed to synthesize novel AgNP/NC composite material. The choice of a cheap and readily available polymer, nitrocellulose, as a stabilizer for AgNPs is a simple method and a step forward in developing bactericidal surface coatings. Results clearly indicate that incorporation of 10 wt% AgNPs into the NC matrix does not negatively affect the chemical and physical properties of NC. The average Ag particle size is around 25 ± 5 nm, and the prepared surface coatings on glass substrates are highly stable under ambient conditions. The antimicrobial and antibiofilm activities of novel composite surface were investigated by the tube method and time-kill kinetic studies, which can be adopted more conveniently in high-throughput experiments. The synthesized material demonstrates good antimicrobial activity and significant inhibition of biofilm development against *P. aeruginosa* (Gram-negative), *S. aureus* (Gram-positive), and *C. albicans*. The time-kill kinetic studies showed that the AgNP/NC composite exhibited strong microbistatic activity against tested microorganisms. The results obtained in this study reveal that AgNP/NC composite material can be used effectively in designing self-disinfecting surfaces, especially for healthcare environments, where hygiene is a priority.

## Figures and Tables

**Table 1:** Calculated rate constants for the reduction of Ag^+^ for the mass ratio (NC : Ag) 1.0 : 0.10 at 40°C and 60°C.

Temperature/°C	*k* × 10^3^/min^−1^	*t*_1/2_/min	*R* ^2^
40	6.00	115.5	0.994
60	14.0	49.50	0.993

**Table 2:** Surface coating properties of clear commercial NC and the prepared AgNPs/NC lacquers.

Parameter	NC lacquer	NC/AgNP lacquer
Viscosity (Krebs)	116	119
Dry to touch time (min)	5-6	5-6
Thinner intake	1 : 1.25	1 : 1.25
Flash-off time (min)	2	1-2

## Data Availability

Data access is available on request through Research Council of the University of Sri Jayewardenepura (https://www.sjp.ac.lk/research/council/). The contact person is Prof. M M Padmala, Chairperson, Research Council, University of Sri Jayewardenepura; the email is pathmalal@sjp.ac.lk.
